# *In Situ* Identification of CD44+/CD24− Cancer Cells in Primary Human Breast Carcinomas

**DOI:** 10.1371/journal.pone.0043110

**Published:** 2012-09-13

**Authors:** Giuseppe Perrone, Laura Maria Gaeta, Mariagiovanna Zagami, Francesca Nasorri, Roberto Coppola, Domenico Borzomati, Francesco Bartolozzi, Vittorio Altomare, Lucio Trodella, Giuseppe Tonini, Daniele Santini, Andrea Cavani, Andrea Onetti Muda

**Affiliations:** 1 Department of Pathology, Campus Bio-Medico University of Rome, Rome, Italy; 2 Experimental Immunology Lab, IDI-IRCCS, Rome, Italy; 3 Department of Surgery, Campus Bio-Medico University of Rome, Rome, Italy; 4 Department of Epidemiology, Campus Bio-Medico University of Rome, Rome, Italy; 5 Senology Unit, Campus Bio-Medico University of Rome, Rome, Italy; 6 Radiotherapy Unit, Campus Bio-Medico University of Rome, Rome, Italy; 7 Oncology Unit, Campus Bio-Medico University of Rome, Rome, Italy; 8 Department of Pathology, Ospedale S. Maria della Misericordia, Udine, Italy; Harvard School of Public Health, United States of America

## Abstract

Breast cancer cells with the CD44+/CD24− phenotype have been reported to be tumourigenic due to their enhanced capacity for cancer development and their self-renewal potential. The identification of human tumourigenic breast cancer cells in surgical samples has recently received increased attention due to the implications for prognosis and treatment, although limitations exist in the interpretation of these studies. To better identify the CD44+/CD24− cells in routine surgical specimens, 56 primary breast carcinoma cases were analysed by immunofluorescence and confocal microscopy, and the results were compared using flow cytometry analysis to correlate the amount and distribution of the CD44+/CD24− population with clinicopathological features. Using these methods, we showed that the breast carcinoma cells displayed four distinct sub-populations based on the expression pattern of CD44 and CD24. The CD44+/CD24− cells were found in 91% of breast tumours and constituted an average of 6.12% (range, 0.11%–21.23%) of the tumour. A strong correlation was found between the percentage of CD44+/CD24− cells in primary tumours and distant metastasis development (p = 0.0001); in addition, there was an inverse significant association with ER and PGR status (p = 0.002 and p = 0.001, respectively). No relationship was evident with tumour size (T) and regional lymph node (N) status, differentiation grade, proliferative index or HER2 status. In a multivariate analysis, the percentage of CD44+/CD24− cancer cells was an independent factor related to metastasis development (p = 0.004). Our results indicate that confocal analysis of fluorescence-labelled breast cancer samples obtained at surgery is a reliable method to identify the CD44+/CD24− tumourigenic cell population, allowing for the stratification of breast cancer patients into two groups with substantially different relapse rates on the basis of CD44+/CD24− cell percentage.

## Introduction

Tumours consist of a heterogeneous cell population, and recent data suggest that a selected group of tumour cells, termed “tumourigenic cancer cells,” bearing stem-like properties such as self-renewal capacity and aberrant differentiation, are capable of giving rise to a wide spectrum of progeny [1]. Although the tumourigenic cancer cells constitute a very small percentage of the total tumour mass, they are believed to be the only subset able to initiate and sustain tumour growth; hence, they are alternatively named “tumour-initiating cells” [2],[3],[4]. Al-Hajj and co-workers were the first to describe a relatively small, phenotypically distinct, subset of cells within human breast cancer. These tumour-initiating cells were distinguished from a substantially larger, non-tumourigenic cell population by the specific cell surface marker phenotype CD44+/CD24− [2]. Since then, the tumourigenic potential of the CD44+/CD24− profile has been repeatedly confirmed in primary tissues [5],[6],[7] and in human breast cancer cell lines [8],[9],[10].

However, the vast majority of these data rely on highly efficient strategies for cell isolation, using flow cytometry in conjunction with *in vivo* analysis. Although *in vitro* experiments and animal models are essential for studying functional differences between defined subsets of cancer cells, there are limitations in the interpretation of these studies [11]. Therefore, validation of the *in vitro/in vivo* findings in clinical samples is of the utmost importance and represents a critical step towards the development of effective, targeted breast cancer treatments.

The identification of human tumourigenic breast cancer cells in surgical samples has recently received attention due to the implications for breast cancer treatment. Current chemotherapy and radiation strategies mainly target actively proliferating cells; CD44+/CD24− cells have been shown to survive cytotoxic therapies due to their slow progression through the cell cycle [12],[13], which represents a likely explanation for treatment failures and recurrences.

The aim of our study was to identify the CD44+/CD24− cell population in surgical specimens of primary breast carcinomas using immunohistochemical methods, with the goal of correlating the amount and distribution of CD44+/CD24− cells with clinicopathological features. Standard immunohistochemical approaches have frequently proved to be unreliable when trying to visualise two or more antigens on the same tissue section, especially when chromogens co-localise to the same cell structure (e.g., the cell membrane). We therefore used fluorochromes with different excitation and emission spectra followed by confocal microscopy analysis to better visualise the distribution and co-localisation of antigens in single tissue sections. In addition, to confirm the reliability and reproducibility of the results obtained by the *in situ* analysis, immunofluorescence and flow cytometry experiments were performed in parallel in a selected number of cases, and the results were compared.

## Results

### CD44 and CD24 analysis using standard immunohistochemistry

Positive immunostaining for CD44 in breast carcinomas was consistently present on the cell membrane of tumour cells and in infiltrating lymphocytes; the latter was therefore selected as an internal positive control. CD24 staining varied substantially among the breast carcinoma cases, as previously described [7],[14]. In some tumours, CD24 was localised predominantly along the plasma membrane, while in others, it was diffusely cytoplasmic.

The identification of the CD44+/CD24− cell population by double immunostaining was performed by determining the presence of Permanent Red membrane staining (CD44+) in the absence of membrane DAB interference (CD24−). The cells with cytoplasmic CD24 staining were also considered CD24 negative because previous functional studies of CD44+/CD24− cells mainly used fluorescence-activated cell sorting (FACS) to assess surface protein expression. The slides were examined by two different investigators (GP and MZ) without knowledge of the corresponding clinicopathological data. Using this approach, conflicting data were obtained in the majority of cancer lesions examined (31/56) because the often variable cytoplasmic and/or membranous distribution of the immune reaction product precluded precise quantification of the percentage of CD24 membrane-positive cells. Consequently, there were substantial differences in the percentage of CD44+/CD24− cells on the same slides.

### CD44 and CD24 analysis by immunofluorescence

**Figure 1:**
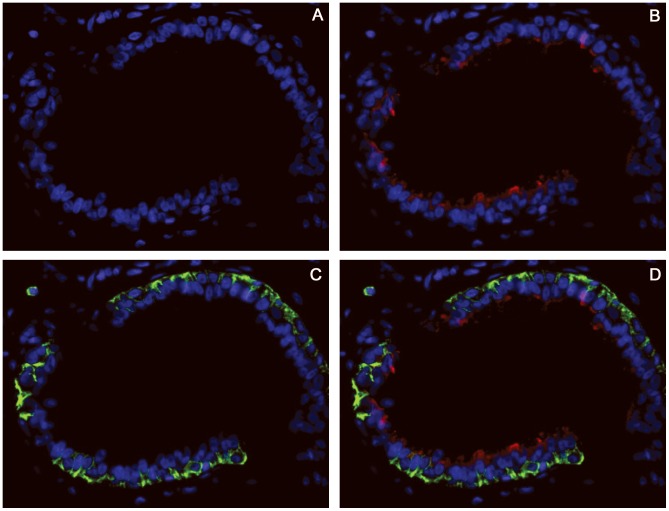
CD44/CD24 expression in normal ductal epithelium. A, DAPI; B, DAPI and CD24 (red); C, DAPI and CD44 (green); D, merged images. Image A shows the double cell layers of breast ductal epithelium composed of basal and luminal cells. Image B shows that CD24 expression is confined to the luminal cell layer, typically in the apical side. Image C shows the typical distribution pattern of the CD44 antigen, which resulted in membrane staining of the basal cell layer and in infiltrating lymphocytes (upper-left corner). D is a merged image of DAPI, CD44 and CD24 images. Original magnification, 400×.

**Figure 2:**
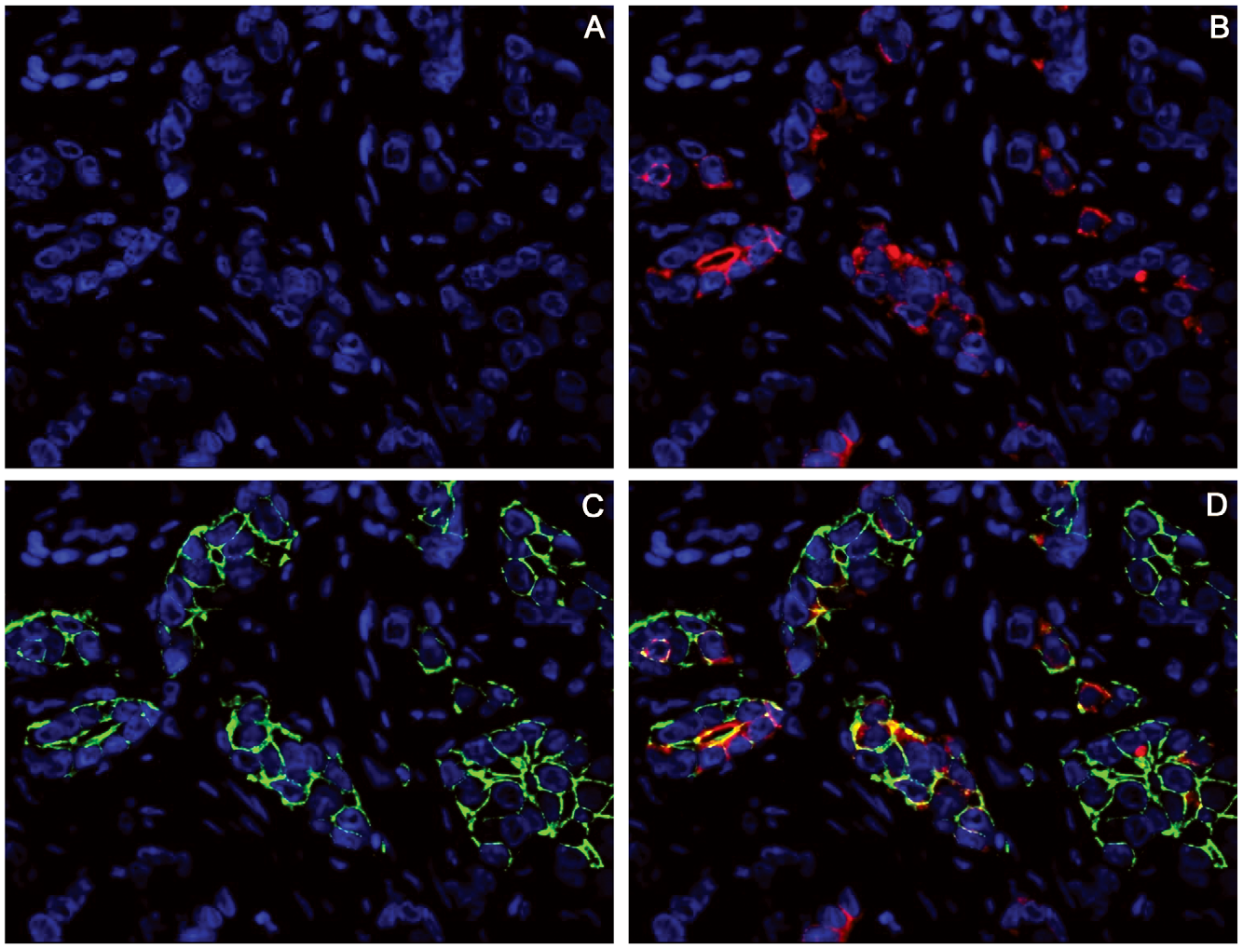
CD44/CD24 expression in human breast cancer. A, DAPI; B, DAPI and CD24 (red); C, DAPI and CD44 (green); D, merged images. The composite image (D) shows the heterogeneity of CD44 and CD24 expression. The breast carcinoma cells displayed four distinct sub-populations of cells based on the membrane expression pattern: CD44+/CD24− cells had green membrane staining without membrane CD24 colocalisation; CD44+/CD24+ cells showed a yellow signal along the cell membrane; CD44−/CD24+ cells showed a red signal for CD24 without CD44 staining; and CD44−/CD24− cells were negative for both antibodies. Original magnification, 400×.

We first analysed the normal breast duct epithelium to confirm that when using immunofluorescence, primary antibodies retained the ability to recognise their specific antigens in paraffin-embedded tissues. Consistent with standard immunohistochemistry, incubation with the CD44 antibody (488 nm, green) resulted in membrane staining of basal cells, while incubation with the CD24 antibody (555 nm, red) produced apical staining of luminal cells (**Figure 1**). Using this method, the breast carcinoma cells displayed four distinct staining patterns on the basis of cell membrane positivity (CD44+/CD24−, CD44+/CD24+, CD44−/CD24+ and CD44−/CD24−). In addition, the merged images showed excellent CD44/CD24 co-localisation (yellow) when present (**Figure 2**). At low magnification, a distinctive distribution of the CD44/CD24 population within the cancer tissue was noted. The CD44+/CD24− cells were typically found in small clusters (10–15 cells), often located along the infiltrating side. Conversely, the CD44−/CD24+ cells were usually found in the central portion of the tumours. Quantification of CD44+/CD24− cell population within each of the 56 breast cancer cases was performed through manual counting of the CD44+/CD24− (green) cells in 10 merged images of representative areas. CD44+/CD24− cells were found in 51/56 (91%) tumours. In these 51 cases, the average fraction of CD44+/CD24− cells was 6.12% (range, 0.11–21.23%).

### Comparison between flow cytometry and immunofluorescence data

The analysis of the results demonstrated that flow cytometry and immunofluorescence found similar percentages of CD44+/CD24− and CD44+/CD24+ cells (**Table 1**), confirming the feasibility of the *in situ* identification of CD44+/CD24− cells. In contrast, significant differences were found in the percentages of CD44−/CD24+ and CD44−/CD24− cells.

**Table 1:** Analysis of the differences* between flow cytometry (FC) and immunofluorescence (IF).

		1	2	3	4	5	
CD44+/CD24−	FC	1.64%	0.54%	0.44%	0%	1.09%	
	IF	2.36%	1.07%	1.09%	0.37%	0.73%	p = 0.599
CD44+/CD24+	FC	3.28%	0.06%	1.86%	0%	0.61%	
	IF	2.14%	1.81%	0.93%	0%	1.49%	p = 0.834
CD44−/CD24+	FC	65.6%	0.16%	30.8%	4.41%	2.25%	
	IF	59.32%	40.87%	33.64%	89.1%	71.36%	p = 0.047
CD44−/CD24−	FC	29.5%	99.2%	66.9%	95.6%	96%	
	IF	36.18%	56.25%	64.34%	10.51%	26.42%	p = 0.047

* Mann-Whitney Test.

### Clinicopathological data

**Figure 3:**
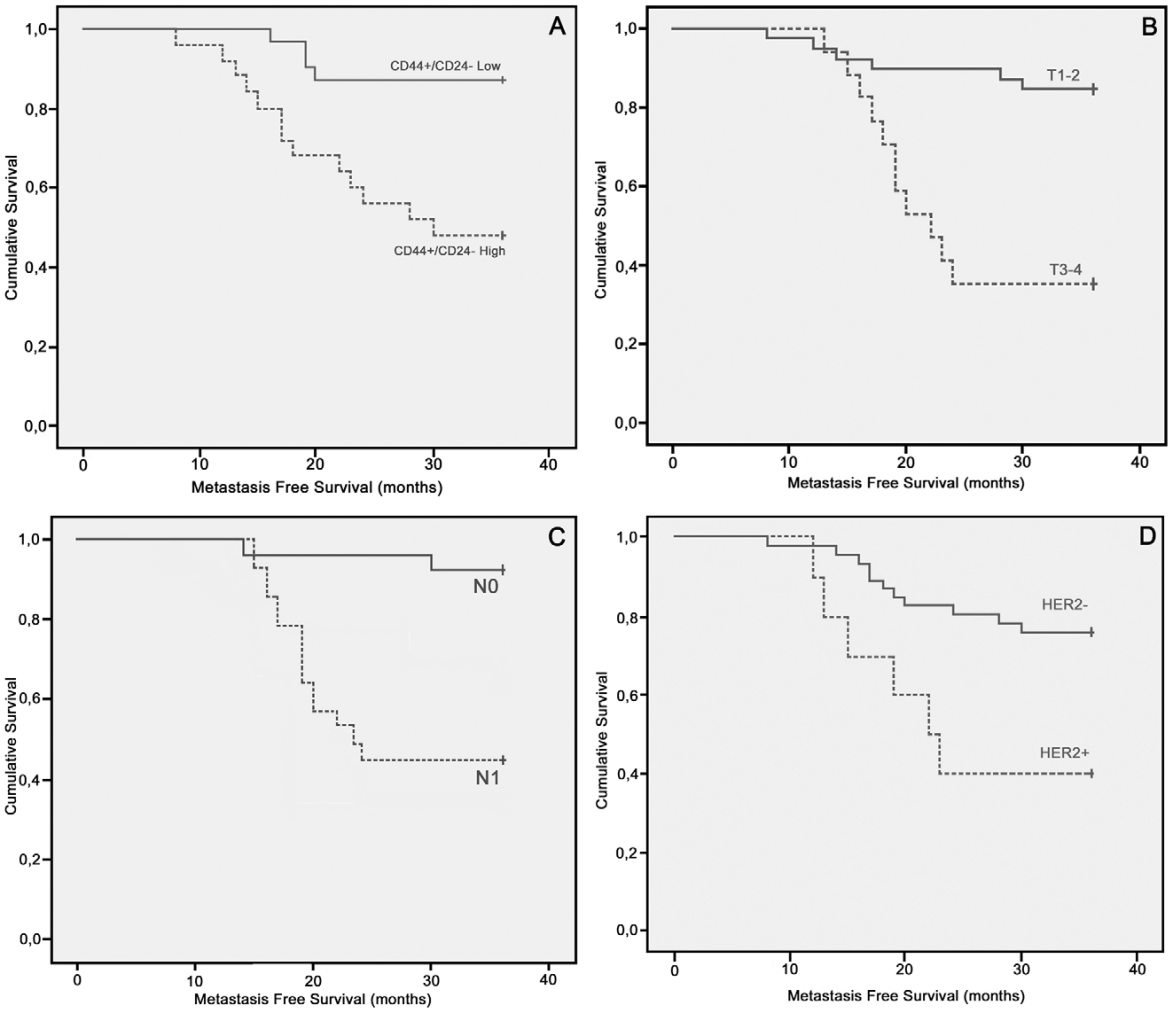
Kaplan–Meier survival plots. Metastasis-free survival in radically resected breast cancer patients according to CD44+/CD24− cells (A), T factor (B), nodal (C) and HER2 (D) status.

To evaluate whether this breast cancer collection (a randomly selected group+a metastatic group) could represent a selection bias in biological behaviour, a correlation analysis (Spearman correlation test) between clinicopathological and immunohistochemical data was performed. Positive correlations were found between tumour size (T), regional lymph node status (N) and distant metastasis (M); between ER and PGR status; and between HER2 status, tumour size (T) and distant metastasis (M). P53 status showed a positive association with tumour size (T) and differentiation grade and a negative correlation with ER and PGR status (**Table 2**). Metastasis-free survival (MFS) was evaluated in univariate and multivariate analyses (**Table 3** and **Table 4**, respectively) with respect to histopathological characteristics and prognostic factors. In the univariate analysis, tumour size (p = 0.001) and HER2 status (p = 0.017) had significant associations with the onset of distant metastasis (**Figure 3**). Using a multivariate Cox regression analysis, tumour size (p = 0.032) was identified as a significant independent factor related to metastasis. The data obtained are in agreement with the literature, suggesting that the breast cancer population presented here does not suffer from selection bias.

**Table 2:** Correlation Matrix* of the clinicopathological and immunohistochemical data.

		T	N	M	G	ER	PGR	KI67	p53	HER2
**%CD44+/CD24−**	r value	0.102	−0.018	**0**.**468**	0.250	**−0**.**406**	**−0**.**438**	0.026	**0**.**320**	0.102
	p value	0.454	0.896	**0**.**000**	0.063	**0**.**002**	**0**.**001**	0.852	**0**.**016**	0.452
**T**	r value		**0**.**417**	**0**.**520**	0.158	−0.097	**−0**.**318**	0.140	**0**.**282**	**0**.**409**
	p value		**0**.**001**	**0**.**000**	0.245	0.477	**0**.**017**	0.303	**0**.**035**	**0**.**002**
**N**	r value			**0**.**280**	0.062	−0.011	−0.124	0.096	0.160	0.137
	p value			**0**.**036**	0.649	0.934	0.361	0.481	0.238	0.313
**M**	r value				0.194	−0.098	−0.213	0.077	0.213	**0**.**301**
	p value				0.153	0.471	0.115	0.571	0.115	**0**.**024**
**G**	r value					**−0**.**538**	**−0**.**443**	**0**.**559**	**0**.**505**	0.220
	p value					**0**.**000**	**0**.**001**	**0**.**000**	**0**.**000**	0.104
**ER**	r value						**0**.**730**	−0.169	**−0**.**428**	−0.221
	p value						**0**.**000**	0.213	**0**.**001**	0.101
**PGR**	r value							−0.084	**−0**.**475**	−0.256
	p value							0.537	**0**.**000**	0.057
**Ki-67**	r value								0.245	0.077
	p value								0.068	0.572
**p53**	r value									0.184
	p value									0.174

* Spearman correlation test: the variables were categorised in the analysis as described in Table 1.

T: tumour size; N: regional lymph nodes; M: distant metastasis; G: differentiation grade.

**Table 3:** Risk of Metastasis (Univariate Analysis).

Variable	Metastasis	
	Hazard Ratio (95% CI)	p value
**CD44+/CD24− (< or >median value)**	5.18 (1.68–15.90)	**0.004**
**Tumour size (T1–2 vs. T3–4)**	5.73 (2.09–15.71)	**0.001**
**Regional lymph nodes (negative vs. positive status)**	2.96 (0.96–9.10)	0.058
**Differentiation Grade (G1–2 vs. G3)**	1.67 (0.64–4.41)	0.290
**Her2 status (negative vs. positive)**	3.39 (1.25–9.23)	**0.017**
**ER status (negative vs. positive)**	0.67 (0.25–1.81)	0.420
**P53 status (negative vs. positive)**	2.47 (0.81–7.61)	0.110
**Ki67 Status (negative vs. positive)**	1.66 (0.38–7.30)	0.500
**Histotype (ductal vs. lobular)**	0.58 (0.17–2.03)	0.390

**Table 4:** Risk of Metastasis (Multivariate Analysis).

Variable	Metastasis	
	Hazard Ratio (95% CI)	p value
**CD44+/CD24− (< or >median value)**	6.00 (1.80–19.95)	**0.003**
**Tumour size (T1–2 vs. T3–4)**	4.43 (1.13–17.28)	**0.032**
**Regional lymph nodes (negative vs. positive status)**	1.43 (0.37–5.40)	0.597
**Her2 status (negative vs. positive)**	1.81 (0.51–6.40)	0.355
**Differentiation Grade (G1–2 vs. G3)**	1.21 (0.31–4.63)	0.778
**ER status (negative vs. positive)**	1.17 (0.33–4.11)	0.800
**P53 status (negative vs. positive)**	1.04 (0.29–3.73)	0.944
**Ki67 Status (negative vs. positive)**	2.17 (0.31–15.17)	0.431
**Histotype (ductal vs. lobular)**	1.07 (0.25–4.49)	0.920

### The clinical significance of the CD44+/CD24− cancer cell population

A strong, positive correlation was found between the percentage of CD44+/CD24− cells and the presence of distant metastasis (p = 0.0001) and p53 expression (p = 0.016); no correlation was found with tumour size (T), regional lymph node status (N), differentiation grade, proliferative index (percentage of Ki67-positive cells) or HER2 amplification status. Moreover, a significant negative association was found with ER and PGR status (p = 0.002 and p = 0.001, respectively; **Table 2**). When considering tumour histotypes, no differences were found between ductal and lobular cancers (p = 0.593); however, significant differences were found when classifying according to breast tumour IHC subtypes (p = 0.018). In particular, luminal-type carcinomas (A and B) showed a significantly lower percentage of CD44+/CD24− cells compared to other immunotypes (**Table 5**).

**Table 5:** Analysis of differences between CD44+/CD24− and breast cancer subtypes.

		n°	% CD44+/CD24− median (range)	p value*
**Histotype**	Ductal	43	5.05 (1.09–9.37)	0.593
	Lobular	13	4.21 (1.04–8.91)	
**IHC Subtype**	Luminal A	35	3.24 (0.50–5.55)	**0.018**
	Luminal B	5	2.44 (1.46–9.48)	
	HER2	5	10.54 (5.80–13.06)	
	Basal-like	11	9.20 (9.09–11.27)	

* Kruscal-Wallis Test.

To establish whether MFS was influenced by the amount of CD44+/CD24− tumour cells, the median value (5.55%) of CD44+/CD24− cells was used as a cut-off value; tumours *below* the median value were categorised as “low”, while those *above* the median value were categorised as “high”. In a univariate analysis, the patients with “high” CD44+/CD24− tumours displayed a shorter median MFS (18 months) than those with “low” CD44+/CD24− tumours (median MFS not reached; p = 0.004; **Figure 3** **and** **Table 3**). Moreover, a multivariate Cox regression analysis identified CD44+CD24− as a significant independent factor related to metastasis (p = 0.003; **Table 4**).

## Discussion

Several *in vitro* and *in vivo* studies indicate that the CD44+/CD24− fraction of breast cancer cells has tumour-initiating properties [2],[10]. Here, we demonstrated that multiple immunofluorescence coupled with confocal microscopy analysis is a simple and reliable method to identify CD44+/CD24− cells in routine surgical breast tumour samples. In addition, the percentage of CD44+/CD24− breast cancer cells is higher in the primary tumours of patients with shorter metastatic-free survival, therefore representing an independent predictor of metastasis development.

Previous functional studies aimed at investigating CD44+/CD24− cells primarily used fluorescence-activated cell sorting (FACS), which assesses cell surface protein expression [2],[10]; we therefore considered the immunostaining to be positive exclusively when it was localised along the cell membrane of cancer cells. Using this approach, conflicting data were obtained when using traditional immunohistochemistry. In particular, the variable pattern of CD24 immunostaining (membrane and cytoplasmic) precluded the precise quantification of the percentage of CD24 membrane-positive cancer cells. Honeth et al., for instance, reported that using standard immunohistochemical methods on breast surgical samples, they could identify a clear variation in the prevalence of CD44+/CD24− tumour cells between tumours of different breast cancer subtypes, although they found no correlation with prognosis [5]. Three additional papers have reported on the immunohistochemical identification of CD44+/CD24− cells in breast cancer surgical samples; however, one group suggested that the prevalence of CD44+/CD24−/low tumour cells may favour distant metastasis [6], while other groups found that the same prevalence was associated with a tendency towards an increase in relapse-free survival of the patient [15],[16]. However, the latter papers specifically stated that membranous staining was not scored distinctly from cytoplasmic staining and was not analysed separately. These results confirmed that traditional (i.e., bright-field based) immunohistochemical methods are frequently unreliable when trying to visualise two or more antigens on the same tissue section and, in particular, when chromogens co-localise to the same cell structure (e.g., the cell membrane).

We therefore shifted to multiple immunofluorescence followed by confocal microscopy, which allows for the unique opportunity to visualise and co-localise two (or more) fluorochromes in tissue samples. A somewhat similar approach was recently used by Snyder et al. [16] who used quantum-dot immunofluorescence and spectral unmixing to evaluate CD44+ and CD24− cells in tissue samples of breast carcinoma; the authors showed that CD44+/CD24− cells have distinctive properties in primary human breast carcinomas in terms of proliferation rate and resistance to chemotherapy.

By studying normal breast tissue, CD24 and CD44 antibodies clearly identified their specific antigens [17],[18],[19], therefore confirming the feasibility and reproducibility of the technique. Breast cancer samples displayed four distinct cell sub-populations based on their membrane expression pattern (CD44+/CD24−, CD44+/CD24+, CD44−/CD24+ and CD44−/CD24−), and merged images showed excellent CD44/CD24 co-localisation, when present. Using this method, we found that CD44+/CD24− cells were typically arranged in small clusters (10–15 cells), often located at the periphery of the tumour, adjacent to the surrounding stroma, while the CD44−/CD24+ cells were usually found in the central portion of the tumour.

A comparison of the results obtained with the *in situ* technique and the FACS method demonstrated that there were no significant differences in terms of CD44+/CD24− and CD44+/CD24+ populations, while significant differences were evident concerning CD44−/CD24− and CD44−/CD24+ cells. A possible explanation for this discrepancy is that in the absence of a marker exclusively localised to the cell membrane (CD44), the variable cytoplasmic CD24 expression may hamper the “in situ” evaluation of cell membrane positivity. In other words, CD44, when present ( = green signal), allows one to distinguish whether CD24 staining is limited to the cytoplasm ( = red signal) or also along the cell membrane ( = co-localisation, yellow signal).

Quantification of CD44+/CD24− cancer cells showed that this population was present in 51/56 (91%) tumours examined; the median population fraction was 6.12% (range, 0.11–21.23%). These figures are similar to ex vivo functional studies performed in animal models [2],[20], although our approach was very different. A multivariate analysis indicated that the percentage of CD44+/CD24− cancer cells was an independent prognostic factor related to metastasis (p = 0.003), suggesting a significant clinical relevance of the CD44+/CD24− subclass of breast cancer cells. This result is in agreement with the “tumourigenic cancer cell” model [2],[10] in which tumours with a larger number of tumourigenic cancer cells may be more likely to metastasise. These data are also consistent with a model in which self-renewing tumourigenic cancer cells represent the cancer seeds, while the tumour microenvironment is the soil that promotes the seed growth [21].

In summary, our results support the strong clinical relevance of the CD44+/CD24− subclass of breast cancer cells. Confocal analysis of fluorescence-labelled samples obtained at surgery will likely allow for the stratification of breast cancer patients into two groups with substantially different relapse rates on the basis of CD44+/CD24− cell percentage. Together, our observations on human breast cancer clinical samples confirm the relevance of previous *in vitro/in vivo* studies and underline the utility of a careful evaluation of the CD44+/CD24− population in naive primary human tumours.

## Materials and Methods

### Tumour samples

Fifty-six breast carcinoma cases were selected from the archives of the Department of Pathology at the Campus Bio-Medico Hospital. The primary selection criterion was the absence of residual disease; all patients were staged before surgery by clinical examination, CT scan of thorax, abdomen, and pelvis and, when indicated, intraoperative ultrasound of the liver. All patients were female, with a median age of 63 y (range, 37–88 y); no patient had received chemo, hormone or radiation therapy before surgery. All patients received conventional postoperative treatment according to their disease. Clinical follow-up was recorded for at least three years after surgery. Metastasis-free survival (MFS) was defined as the time elapsed between excision of the primary tumour and manifestation of metastasis. To evaluate the biological features of aggressive cancers, 16 patients were specifically selected on the basis of the development of distant metastasis during the first three years of follow-up. The pathological findings were obtained from the original pathology reports. In addition, tumour–node–metastasis status classification was reassessed according to AJCC [22]. The combined histological grade (1, 2, and 3) of infiltrating ductal carcinomas was obtained according to a modified Scarff-Bloom-Richardson histological grading system with guidelines as suggested by Nottingham City Hospital pathologists [23]. The clinicopathological features are summarised in **Table 6**. The study was approved by the Campus Bio-Medico University Ethics Committee (project: “Tumourigenic cells in breast and pancreas cancer”). Informed written consent was obtained from all patients.

**Table 6:** Patient characteristics.

	Randomly selected group	Metastatic group	Total
**# of cases**	40	16	56
**Median age (range)**	64 (38–88) years	58 (37–77) years	63.5 (37–88) years
**Tumour size (T)**			
T1	25 (62.5%)	1 (6.2%)	26 (46.3%)
T2	9 (22.5%)	4 (25.0%)	13 (23.3%)
T3	1 (2.5%)	2 (12.5%)	3 (5.4%)
T4	5 (12.5%)	9 (56.3%)	14 (25.0%)
**Regional lymph nodes (N)**			
Negative	21 (52.5%)	4 (25.0%)	25 (44.6%)
Positive	19 (47.5%)	12 (75.0%)	31 (55.4%)
**Distant metastasis (M)**			
Negative	39 (97.4%)	0(0.0%)	39(69.6%)
Positive	1 (2.6%)	16(100%)	17(30.4%)
**Grade**			
Well-differentiated	9 (22.5%)	0 (0.0%)	9 (16.0%)
Moderately differentiated	12 (30.0%)	7 (43.7%)	19 (34.0%)
Poorly differentiated	19 (47.5%)	9 (56.3%)	28 (50.0%)
**Histotypes**			
Ductal	30 (75.0%)	13 (81.2%)	43 (76.8%)
Lobular	10 (25.0%)	3 (18.8%)	13 (23.2%)
**IHC subtypes**			
Luminal A	27 (67.5%)	8 (50.0%)	35 (62.5%)
Luminal B	3 (7.5%)	2 (12.5%)	5 (9.0%)
HER2+	1 (2.5%)	4 (25.0%)	5 (9.0%)
Basal-like	9 (22.5%)	2 (12.5%)	11 (19.5%)

### Definition of breast tumour subtypes

The breast tumour subtypes were defined according to Carey et al. [24] as follows: luminal A (ER+ and/or PR+, HER2−), luminal B (ER+ and/or PR+, HER2+), HER2+ (ER−, PR− and HER2+) and basal-like (ER−, PR−, HER2−, cytokeratin 5/6 positive, and/or HER1+). ER and PR were considered positive when nuclear staining was present in 10% or more of the tumour cells; HER1, HER2 and basal cytokeratin (cytokeratin 5/6) positivity was graded according to previously established and published criteria [25]. To determine HER2 gene amplification, the ZytoLight SPEC HER2/CEN 17 Dual Colour (ZytoVision) FISH assay was performed and evaluated as described previously [26].

### Immunohistochemistry for CD44/CD24

Double immunohistochemical staining was performed on 3-µm-thick paraffin-embedded tissue sections using an HRP and ALP micro-polymer detection kit (Double Stain Polymer Detection Kit #2, Biocare Medical, CA, USA). The sections were incubated with a primary antibody cocktail containing a mouse polyclonal anti-CD44v6 antibody (clone VFF18, 1∶200, Millipore, Billerica, MA, USA) and a mouse monoclonal anti-CD24 antibody (clone SN3b, Thermo Fisher Scientific, UK). The antibodies have been previously used and validated (CD44 [17],[27],[28],[29]; CD24 [5],[15],[17]).

Enzymatic activity was detected using DAB (Dako, Denmark) for 5 minutes followed by Fast Red (Vulcan Fast Red Chromogen Kit, Biocare Medical, CA, USA). Negative control slides processed without primary antibody were included for each staining. Ductal epithelial cells were used as a positive internal control for CD24 (luminal cells) and CD44 (basal cells) expression. The immunostaining evaluation was performed at high power (400×) in multiple representative fields to calculate the percentage of immunoreactive cells in a total of at least 1,000 neoplastic cells. The quantification of the CD44+/CD24− population was therefore performed by considering “positive” only the tumour cells with Fast Red membrane staining in the absence of DAB staining.

### Immunofluorescence analysis

Consecutive 3-µm sections were cut from each block for immunofluorescence experiments. Incubation with primary antibodies against CD44 and CD24 was followed by Alexa fluor 488-conjugated anti-mouse or anti-rabbit IgG (H+L) and Alexa fluor 555-conjugated anti-mouse or anti-rabbit IgG (H+L; Invitrogen, USA). A mounting medium containing DAPI (Vectashield, Vector Laboratories, USA) was used. Negative control slides processed without primary antibodies were included for each staining. All sections were examined with a Zeiss LSM 510 Meta confocal laser-scanning microscope (CLSM). The optical sections (i.e., the images obtained from laser scans of subsequent x-y planes at various z-positions in the specimen) were collected from at least 10 high-power fields in each case. Briefly, for double-labelled specimens, the dual channel mode was employed, and the sections were scanned simultaneously at both wavelengths (488/514 and 514/647, respectively) with the laser intensity, confocal aperture, gain and black level settings kept constant. All scans were performed using the same objective (63× oil immersion objective; magnification 630×). The cell counts were performed in representative areas of collected composite images (at least 10 fields for each case at high magnification) in which the signal from the fluorochrome had been assigned a different pseudo-colour (green for 488, red for 555 and blue for DAPI). Approximately 1000 cells were counted for each sample. Only the tumour cells with CD44 membrane staining (green) without membrane localisation or colocalisation of CD24 (red or yellow, respectively) were considered positive.

### Flow Cytometry

To confirm the reliability of the “in situ” method to identify the CD44+/CD24− cancer cell population, we performed in parallel flow cytometric experiments and immunofluorescence analysis in 5 consecutive breast cancers. To perform flow cytometry analysis, fresh cancer tissue was used. The human breast cancer tissues were mechanically minced into small pieces and dissociated with 250 U/ml collagenase type III (Sigma) in RPMI 1640 medium supplemented with 100 U/ml penicillin and 100 mg/ml streptomycin (all from Lonza, Basel, Switzerland) at 37°C for 3–4 hours. Depletion of leukocytes was accomplished using CD45 microbeads (Miltenyi Biotec, Germany). The cell suspension was filtered through a 70-mm nylon cell strainer and washed once with RPMI with 20% foetal bovine serum (FBS; Hyclone, Logan, UT). Freshly single cells were then washed twice with phosphate-buffered saline (PBS) containing 2% human serum (HS; Sigma-Aldrich, St. Louis, MO) and subjected to surface marker profiling analysis. The following antibodies were used: anti-EpCAM-PerCP-Cy5.5 (clone EBA-1, IgG1), anti-CD44-APC (clone G44-26, IgG2b), anti-CD45-V450 (clone 2D1, IgG1), and anti-CD24-PE (clone ML5, IgG2a; all from BD Bioscience, San Diego, CA). In addition, mouse IgG isotype controls were purchased from BD Bioscience. Single cells were stained with directly labelled antibodies for 20 minutes on ice in the dark in PBS containing 1% HS. Staining with the matched isotype control Ig was included. The cells were analysed on a BD FACS ARIA. Sample analysis was performed using BD FACS Diva software.

### Statistical analyses

Spearman's rank correlation test (two-sided) was used to assess relationships between immunohistochemical and clinicopathological parameters. Overall survival and metastasis-free survival in the two groups were analysed and compared by the Kaplan–Meier method. The significance of the differences in survival distribution among the prognostic groups was evaluated by the Cox proportional hazards model applied to the univariate and multivariate survival analysis. A P value<0.05 was regarded as significant in two-tailed tests. STATA Software (version 8.00, StataCorp LP, College Station, Texas, USA) was used for statistical analyses.
